# Health Impacts of the COVID-19 Pandemic Among Older Adults With Chronic Conditions

**DOI:** 10.1093/geroni/igab046.2738

**Published:** 2021-12-17

**Authors:** Courtney Polenick, Vanessa Lee, Shreya Salwi, Nikita Daniel, Annie Zhou, Summer Meyers, Diarratou Kaba

**Affiliations:** 1 University of Michigan, Ann Arbor, Michigan, United States; 2 University of Michigan, University of Michigan, Michigan, United States

## Abstract

The COVID-19 pandemic may have adverse health implications, particularly among older adults with chronic conditions who are at increased risk of severe illness. This cross-sectional study examined the early health impacts of the pandemic among adults aged 50 and older with chronic conditions. Participants included 700 adults (M = 64.60 years, SD = 8.85, range = 50 – 94) from Michigan (82.4%) and 33 other U.S. states who reported at least one chronic condition and completed an anonymous online survey between May 14 and July 9, 2020. Of these, 488 also provided open-ended responses. Individuals reported lower illness self-efficacy, less consumption of fruits, vegetables, and fried foods, and lower physical activity, along with greater alcohol use since the pandemic. About half (42.7%) reported worsened sleep. One in five (20.1%) reported at least some difficulty obtaining medications and over half (60.4%) reported at least some difficulty receiving routine care. Almost two-thirds (63.9%) had delayed preventative care and one in five (19.3%) had delayed essential medical treatment. Nearly half (42.6%) avoided contacting care providers about a physical health concern and one in eight (12.9%) avoided reporting mental health concerns. Qualitative data revealed that the pandemic has influenced how participants cared for their physical health through following guidelines related to COVID-19; coping with daily routine changes; greater awareness of self-care; mental health impacts; and health care disruptions. Older adults with chronic conditions report distinct pandemic-related challenges for self-care and health care that should be addressed in interventions to maintain their health and functioning.

